# I Phillips

**Published:** 1889-05

**Authors:** 


I PHILLIPS, our active and accommodating business man in the Surgical Instrument line, was the party who furnished the unique and beautiful Drug Case which was presented at the Southern Medical College Commencement, as a prize for the best examination in physiology, It contained in addition to the usual number of vials, etc., an outfit for hypodermic medication. Mr. Phillips has greatly enlarged his business, and can now supply anything needed in the line of instruments and appliances for the use of medical practitioners. His advertisement may be seen on page 9 of this Journal.

